# Ultrasonographic evaluation of fibrous structure and mechanical characteristics of the infrapatellar fat pad in female patients with knee osteoarthritis: a cross-sectional study

**DOI:** 10.1186/s12891-026-10250-z

**Published:** 2026-07-23

**Authors:** Syoya Nakanishi, Masahiro Tsutsumi, Takashi Kitagawa, Toshinori Miyashita, Kei Narui, Makoto Wada, Shintarou Kudo

**Affiliations:** 1 Graduate School of Health Sciences, Morinomiya University of Medical Sciences, 1-26-16 Nankoukita, Suminoe-ward, Osaka, 559-8611 Japan; 2 Wada Orthopedic Clinic, Osaka, Japan; 3 Inclusive Medical Sciences Research Institute, Morinomiya University of Medical Sciences, Osaka, Japan; 4 Department of Rehabilitation, Higashiosaka Hospital, Osaka, Japan; 5 Osaka Bay Clinic, Morinomiya University of Medical Sciences, Osaka, Japan; 6 AR-Ex Medical Research Center, Tokyo, Japan

**Keywords:** Knee osteoarthritis, Infrapatellar fat pad, Fibrous, Ultrasonography

## Abstract

**Background:**

Fibrosis of the infrapatellar fat pad (IFP) is common in knee osteoarthritis (KOA); however, a comprehensive evaluation is lacking. This study aimed to use ultrasonography to investigate the fibrous structure and mechanical characteristics of the IFP in patients with KOA.

**Methods:**

This cross-sectional study included patients with KOA, age-matched controls, and healthy young adults. Fifty knees with KOA were analyzed for Kellgren–Lawrence grade differences, whereas 50 knees with KOA, 14 age-matched controls, and 15 knees from healthy young adults were analyzed for between-group comparisons. The primary outcomes were IFP shear wave stiffness, fiber orientation intensity, proportion of high-intensity area, and movement, assessed using shear wave elastography, ImageJ analysis, binarized US analysis, and particle image velocimetry, respectively. The association between magnetic resonance imaging (MRI) signal intensity and outcomes was evaluated in the KOA group.

**Results:**

Compared with the controls, the KOA group demonstrated greater IFP stiffness (95% confidence interval [CI] [0.152, 0.469]) and proportion of high-intensity area (95% CI [2.737, 11.263]) with reduced movement of the superficial part (95% CI [-0.240, -0.104]) and the deep part (95% CI [-0.290, -0.131]). MRI signal intensity of the IFP was positively correlated with the proportion of high-intensity area among the 14 KOA knees with MRI data (*r* = 0.720, *p* = 0.004). The proportion of high-intensity area in grade IV was significantly higher than that in grades I and II.

**Conclusion:**

IFP in KOA exhibits distinct ultrasonographic characteristics, including increased stiffness and proportion of high-intensity area, along with restricted movement. Notably, the proportion of high-intensity area was significantly higher in KL grade IV than grades I and II, and correlated with MRI signal intensity, suggesting its potential utility as a quantitative ultrasound-derived imaging parameter for assessing IFP tissue alteration in KOA.

**Supplementary Information:**

The online version contains supplementary material available at https://doi.org/10.1186/s12891-026-10250-z.

## Background

Inflammation and subsequent fibrosis of the infrapatellar fat pad (IFP) are common characteristics in patients with knee osteoarthritis (KOA) and synovitis [1, 2]. The IFP comprises adipose tissue and, together with the synovial membrane, constitutes an anatomo-functional unit [3]. The IFP disperses mechanical stress across the anterior knee. In KOA, chronic inflammation leads to adipose tissue remodeling and fibrosis, which alters its biomechanical properties, consequently impairing its stress-dispersing function and causing joint damage [4]. Therefore, elucidating the pathogenesis of the IFP is important for understanding the progression of KOA.

Magnetic resonance imaging (MRI) is currently the gold standard for assessing IFP pathology, particularly for detecting signal-intensity changes associated with synovitis, neovascularization, and edema (such as Hoffa-synovitis) [5–10]. Alterations in MRI-based signals are associated with KOA progression and pain severity [11, 12]. While MRI is a reference standard for these pathological changes, recent ultrasonographic studies have also highlighted associations between IFP parameters, such as stiffness [13–15], kinematics [16–18], and echogenicity [19, 20], and KOA severity or symptoms. However, to understand the interrelated pathological changes of the IFP in KOA, a comprehensive multiparametric assessment is needed.

Inflammation-induced fibrosis in adipose tissue is characterized by interrelated structural and mechanical changes [21]. Ultrasonography is promising for evaluating four distinct but interrelated parameters: proportion of high-intensity area (reflecting the proportion of connective tissue), shear wave elastography-derived stiffness (the mechanical consequence of fibrosis) [13–15], movement (the functional consequence of tissue stiffening) [22–24], and fiber orientation intensity (indicating structural disorganization) [25]. These multiparametric assessments may therefore enable the quantitative evaluation of fibrous structural changes and associated mechanical characteristics of the IFP in patients with KOA. It is essential to distinguish disease-specific alterations from normal aging to interpret the pathological changes. Therefore, this study included an age-matched older control group and a healthy young group to establish a baseline for normal IFP structure and mechanics.

This study aimed to use ultrasonography to investigate the fibrotic and mechanical characteristics of the IFP in KOA by assessing the proportion of high-intensity area, fiber orientation intensity, stiffness, and movement. Since chronic inflammation induces progressive fibrosis and tissue stiffening in the IFP, we hypothesized that the KOA group would exhibit distinct quantitative alterations, specifically, increased stiffness and proportion of high-intensity area, along with restricted movement, compared with the age-matched control and young groups. Furthermore, we hypothesized that these ultrasound-derived parameters would correlate with MRI signal intensity and differ across radiographic disease severity (Kellgren–Lawrence [KL] grades).

## Materials and methods

### Study design and setting

This cross-sectional study aimed to clarify the characteristics of ultrasonographic findings of the IFP in patients with KOA. Data were collected from an orthopedic clinic in Hirakata, Osaka, Japan, between November 2023 and March 2024.

### Participants

Participants in the KOA group met the following inclusion criteria: female sex, age 50–80 years, and orthopedically diagnosed unilateral or bilateral KOA based on the KL grading system [26]. Only female participants were included due to a higher prevalence of KOA in women, potential for a skewed sex ratio in a mixed cohort, and the need to eliminate the confounding effects of sex differences on adipose tissue characteristics. As KL grade 1 is considered an early stage of KOA [27], it was included in the KOA group. Participants were required to report anterior or medial knee pain on the examined side when walking or climbing stairs. The side with the most knee pain was considered the test side for bilateral KOA. The control group included age-matched female participants and healthy older individuals without knee pain who had never previously been diagnosed with KOA. The inclusion criterion for the young age group was healthy young adults without knee pain at rest or during movement. Exclusion criteria for all groups were: history of knee surgery, knee trauma, or neurological disease, cancer, inflammatory diseases, rheumatologic diseases, or systemic disorders.

The study protocol was approved by the Ethics Committee of Morinomiya University of Medical Sciences (Osaka City, Osaka, Japan (approval number 2023 − 124). In accordance with the Declaration of Helsinki, all participants received a full explanation of the study purpose, methods, data management, and use of the results, and provided written informed consent prior to participation.

### Ultrasound imaging protocol

**Fig. 1:**
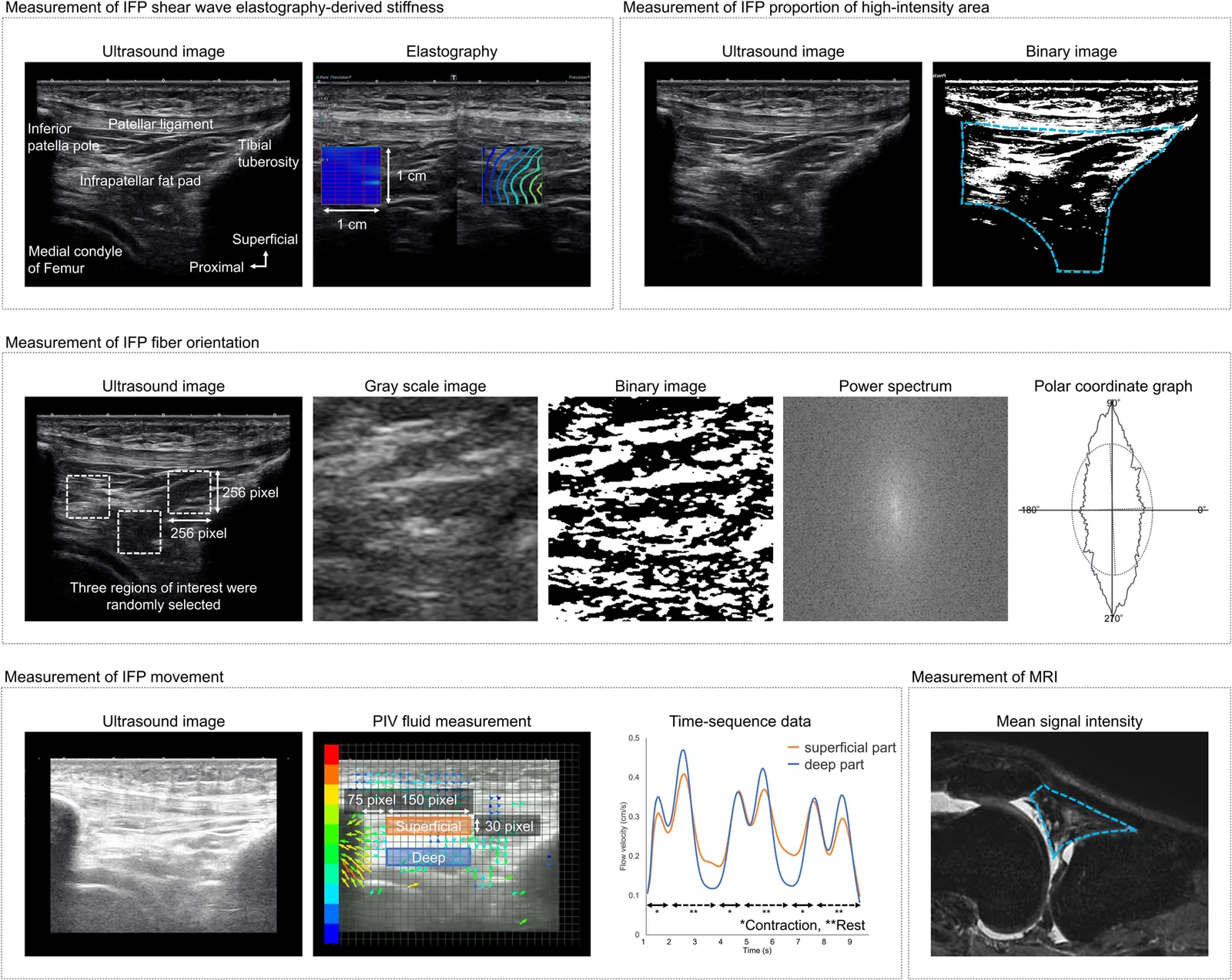
Schematic illustration of the measurement and image analysis procedures. Representative images demonstrating the quantitative assessment of the IFP. The figure illustrates the measurement processes for IFP shear wave elastography-derived stiffness, proportion of high-intensity area via image binarization, fiber orientation intensity via power spectrum analysis, IFP movement via particle image velocimetry (PIV), and MRI signal intensity

Participants were positioned in a long sitting position with the ankle joint secured using a belt. A manchette of a blood pressure cuff (FC-100, FOCAL CORPORATION Inc., Chiba, Japan) was placed in the popliteal space and pressurized to 100 mmHg with 10° knee flexion, following our previously established protocol [28]. This pressure was chosen because it allowed stable measurements while standardizing the intensity of muscle contraction during the task, and avoided alterations in the knee flexion angle, which could affect IFP kinematics. We used an 18-MHz transducer (PLI-1205BX, Canon Medical Systems Inc., Tochigi, Japan) connected to an ultrasound imaging system (Aplio i700; Canon Medical Systems, Inc., Tochigi, Japan). The transducer was aligned along the long axis of the patellar ligament. The IFP was visualized using anatomical landmarks, including the inferior patellar pole, medial femoral condyle, and the tibial tuberosity (Fig. 1). A single physical therapist performed all ultrasound imaging and subsequent analyses. For measurements during rest, one representative static image per participant was captured. Measurements of IFP stiffness, fiber orientation, and proportion of high-intensity area were obtained at rest using the following ultrasonographic conditions: gain, 80; dynamic range, 60; frame rate, 43 frames per second; and imaging depth: 3.5 cm. The IFP movement was assessed during the exercise task described below with the following conditions: gain, 100; dynamic range, 60; frame rate, 43 frames per second; and imaging depth, 3.5 cm. During all acquisitions, the time-gain compensation was kept neutral, and the focal zone was set at the depth of the IFP. The examiner carefully applied minimum probe pressure to the skin to avoid deforming the superficial tissues and the IFP.

### Measurement of IFP shear wave elastography-derived stiffness

IFP stiffness was measured using shear wave elastography (Aplio i700, Canon Medical Systems Inc., Tochigi, Japan) (Fig. 1). Regions of interest measuring 1 × 1 cm were set within the IFP, and the average of the three regions of interest was used as the representative measure. This method has demonstrated high reliability (> 0.80) in previous studies [13].

### Measurement of IFP fiber orientation

Ultrasonographic images were analyzed using ImageJ software (version 1.54 g; National Institutes of Health, Bethesda, MD, USA) (Fig. 1). Three [256 × 256-pixel] regions of interest were randomly selected within the IFP. The selected images were analyzed, and fiber orientation intensity was calculated using Fiber Orientation Analysis Version 8.13 [29]. The orientation intensity values are ≥ 1, with higher orientation intensity indicating that the fibers in the region of interest are oriented in the same direction [30].

### Measurement of IFP proportion in the high-intensity area

Ultrasound images were binarized in ImageJ to correct for light and dark irregularities, using thresholds of 60 (lower) and 255 (upper). These threshold values were determined based on a preliminary pilot study to isolate high-intensity connective tissue optimally. Regions of interest were manually delineated along the outer edge of the IFP (Fig. 1). The area of interest was calculated as the sagittal area of the IFP. Subsequently, the high-intensity US area in the region of interest was calculated. The IFP proportion of the high-intensity area was calculated as shown in Eq. 1.

1 $$\begin{aligned}&\text{IFP proportion of high-intensity area} =\\&\left(\text{area of the high-intensity area in the region of interest/area of the IFP}\right)\\&\times100\end{aligned}$$

### Measurement of IFP movement

Participants performed isometric quadriceps exercises (IQE) as strongly as possible by pressing the popliteal space against a manchette pressurized to 100 mmHg. Based on previous studies [28, 31], 50% intensity of maximal IQE is the exercise intensity, which is calculated using Eq. 2.

2 $$\mathrm{IQE}=(\text{maximum IQE pressure} [\mathrm{mmHg}]-100[\mathrm{mmHg}])\times0.5$$

The exercise task was a 50% intensity IQE consisting of three repetitions of 1-s contractions, followed by 2-s rest periods. All participants performed IQE at 50% intensity with visual feedback using a sphygmomanometer. The video was analyzed using particle image velocimetry (PIV) fluid measurement software (Library, Tokyo, Japan; tracking pixels 15 × 21; two-frame interval, time interval: approximately 0.023 s). Pixel-to-distance was calibrated using the depth scale displayed on the ultrasound images. Regions of interest were defined for both the superficial and deep IFP regions based on previous studies [22]. The vertical width of the superficial part was 30 pixels from the deep edge of the patellar ligament, with a lateral width of 150 pixels distal from a point 75 pixels distal to the inferior patellar pole. The vertical width of the deep part was 30 pixels from the high-intensity septum at the center of the IFP. The vertical width was assumed to be the same as that of the superficial part. The flow velocities of the superficial and deep parts of the IFP were arranged using time-sequence data. The magnitude of the resultant velocity vector was used for analysis. The contraction phase was identified using distinct peaks appearing in the flow velocity time-series data, corresponding to the 1-s contraction tasks (Fig. 1). The maximum flow velocity (cm/s) of the IFP during contraction was calculated for both the superficial and deep parts, and the mean of the three trials was used as the representative value.

### MRI protocol

Knee MRI was performed using a non-contrast 3.0 T whole-body MRI system (Discovery 750 W; GE Healthcare Japan Inc., Tokyo, Japan). Fat-suppressed T2-weighted sagittal images were used. The MRI sequence parameters were as follows: three-dimensional CUBE T2-weighted fat-suppressed, variable flip angles, repetition time 1742 ms, echo time 42.92 ms, window width/window level 2825/1053, and slice thickness of 0.6 mm to obtain 428 slices with no inter-slice gap. One sagittal slice clearly depicting both the patellar ligaments was selected for analysis. Regions of interest were manually drawn around the outer border of the IFP. Signal intensity, serving as a semi‑quantitative measure, was evaluated using histogram analysis in ImageJ to obtain the average signal intensity of a specific area. Based on previous studies [5], the mean signal intensity of the selected sagittal slices was used for the analysis (Fig. 1).

### Reliability

The reliability of the IFP fiber orientation, proportion of high-intensity area, and movement measurements was examined using 10 knees from five healthy adults with no history of knee joint disease (five females, mean age 28.4 ± 8.0 years, height 158.4 ± 3.5 cm, weight 47.8 ± 3.1 kg), and 10 patients with KOA (separate participants recruited under the same eligibility criteria as in this study; mean age 67.1 ± 9.1 years, height 156.9 ± 4.1 cm, and weight 58.8 ± 6.2 kg). Measurements were performed by two physical therapists with 3–5 years of experience in performing ultrasonography. Both examiners conducted measurements on the same day, and one examiner repeated the measurements 7 days later.

### Study size

We used pilot study data on the IFP proportion in high-intensity areas for each of the five patient groups to estimate the sample size for between-group comparisons (KOA, older adults, and young age groups). A priori power analysis (effect size of 0.70, α error of 0.05, and power of 0.8) based on analysis of covariance (ANCOVA) was conducted. The analysis yielded a required total sample size of 36.

We used a pilot study of the IFP proportion in high-intensity areas for each group of five patients to estimate the sample size. For comparisons among KL grades I, II, III, and IV of the KOA group, a priori power analysis (effect size of 0.78, α error of 0.05, and power of 0.8) based on ANCOVA was conducted. The analysis yielded a required sample size of 36. G*Power version 3.1 was used for all priori power analyses.

### Statistical methods

#### Reliability

The IFP fiber orientation, proportion of high-intensity area, and movement were calculated using the intra-rater correlation coefficient (ICC 1.3) and inter-rater correlation coefficient (ICC 2.3). Measurement error was assessed using the standard error of the measurement (SEM) and the minimal detectable change at the 95% confidence interval (MDC95). SEM was calculated using Eq. 3 [18, 22].

3 $$\mathrm{SEM}=\text{standard deviation}\times\;\surd(1-\mathrm{ICC})$$

MDC was calculated using Eq. 4.

4 $$\mathrm{MDC}95=1.96\times\;\surd2\times\mathrm{SEM}$$

#### Between-group ultrasonographic imaging analysis

A between-group ultrasound analysis set (KOA, *n* = 50; control, *n* = 14; young, *n* = 15) was used for between-group comparisons. Separate ANCOVA was performed to compare the five primary outcomes: IFP stiffness, fiber orientation, proportion of high-intensity area, superficial movement, and deep movement. Body mass index (BMI) was included as a covariate for all analyses. Maximum IQE pressure was additionally included as a covariate for superficial and deep movement to adjust for differences in relative exertion. Age was not included as a covariate in this analysis because the objective was to evaluate the effects of age differences among the groups. Post-hoc comparisons were conducted using the Tukey–Kramer test. A Bonferroni correction was applied, setting the threshold for statistical significance at 0.01 (0.05/5) to account for multiple comparisons among the five primary outcomes.

#### Exploring the relationship between ultrasound imaging and MRI imaging analysis

Using the KOA MRI-correlation analysis set (*n* = 14), the relationship between IFP MRI signal intensity and IFP stiffness, fiber orientation, proportion of high-intensity area, superficial movement, and deep movement was examined using Spearman’s rank correlation coefficients. A Bonferroni correction was applied, with the threshold for statistical significance set at 0.01 (0.05/5) to adjust for multiple testing across the five correlation analyses.

#### Ultrasound imaging analysis of the between-KL grade difference

As an exploratory analysis, using the KOA KL-grade analysis set (*n* = 50), a separate ANCOVA was used to compare IFP stiffness, fiber orientation, proportion of high-intensity area, superficial movement, and deep movement among KL grades I, II, III, and IV in the KOA group. Age and BMI were included as covariates for all analyses. Maximum IQE pressure was additionally included for movement analyses. The assumptions of ANCOVA were verified. Before ANCOVA, the assumptions of normality, homoscedasticity, and homogeneity of regression slopes were confirmed. Post-hoc tests were performed using the Tukey–Kramer test. Statistical significance was evaluated using the Bonferroni-adjusted alpha level of 0.01. All statistical analyses were performed using Jamovi (version 2.6.44.0; The Jamovi Project, 2024, Sydney, Australia).

## Results

### Participants

**Fig. 2:**
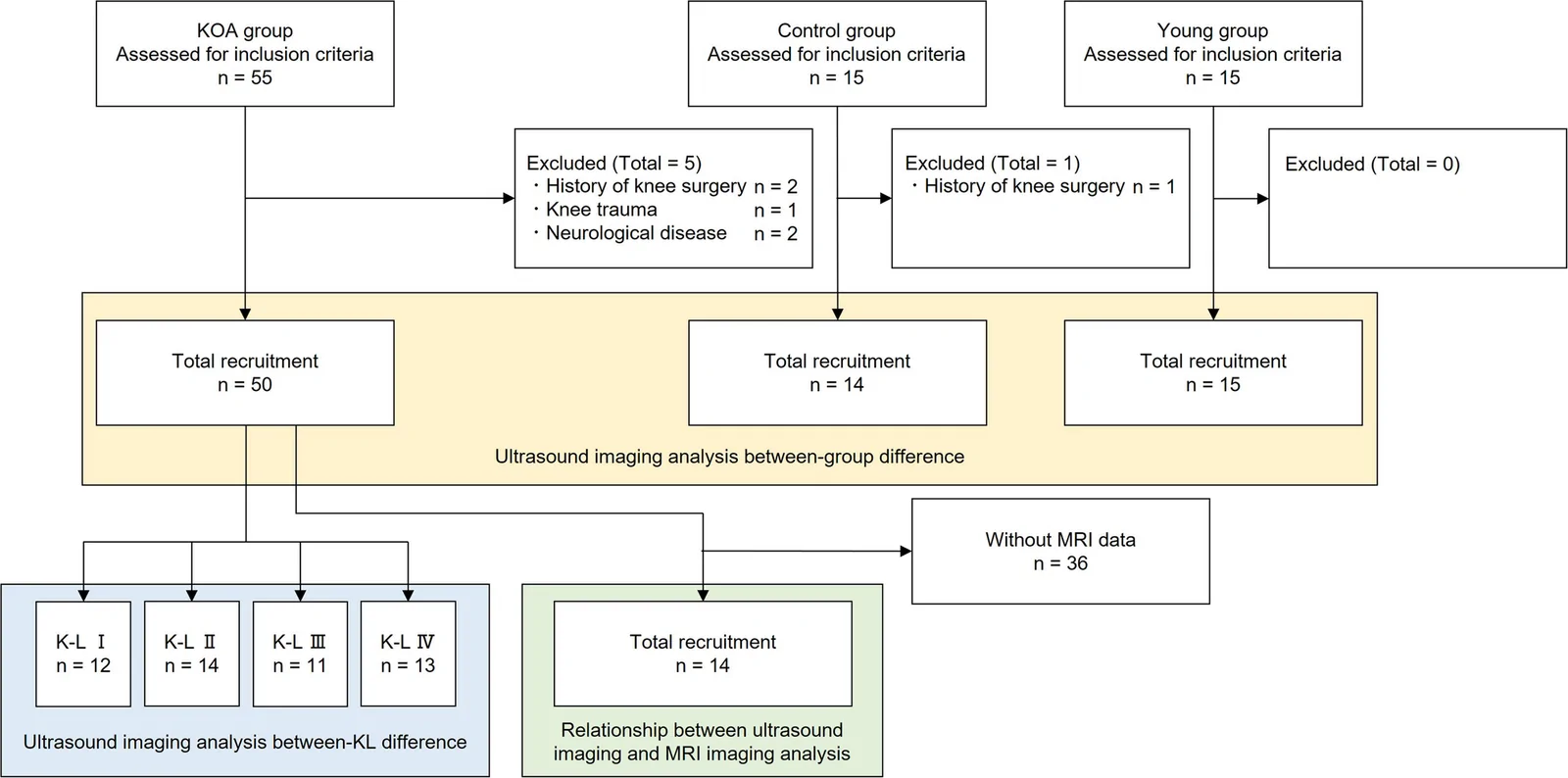
Flow chart of the eligibility criteria KOA, knee osteoarthritis; KL, Kellgren-Lawrence grade; MRI, magnetic resonance imaging

Table 1 summarizes the participants’ characteristics, and Fig. 2 shows a flowchart of the participant recruitment and analysis sets. Fifty-five knees met the inclusion criteria for the KOA group; five knees were excluded. Finally, 50 knees were used for the between-group ultrasound imaging analysis (KOA group, *n* = 50; control group, *n* = 14; young age group, *n* = 15) and the ultrasound imaging analysis for KL grade differences (K-L I, *n* = 12; K-L II, *n* = 14; K-L III, *n* = 11; K-L IV, *n* = 13). Furthermore, 14 of the 50 knees with available MRI data were included in the analysis of the relationship between ultrasound and MRI imaging. In the control group, 15 knees met the inclusion criteria; one knee was excluded, leaving 14 knees for analysis. In the young age group, 15 knees met the inclusion criteria, and none met the exclusion criteria. Finally, all eligible participants (KOA group, *n* = 50; control group, *n* = 14; young age group, *n* = 15) were included in the analysis of between-group differences.

**Table 1:** Characteristics of participants

	Young group	Control group	KOA group	KOA group				Between group	Between K-L
				K-L Ⅰ	K-L Ⅱ	K-L Ⅲ	K-L Ⅳ	*P*-Value	*P*-Value
n	15	14	50	12	14	11	13		
Age (years)	19.8 (2.8)	67.1 (3.8)	66.6 (11.4)	58.3 (8.8)	63.6 (10.8)	71.6 (8.0)	73.1 (10.7)	< 0.001	0.003
Height (cm)	158.7 (6.1)	158.5 (5.9)	155.1 (5.3)	156.9 (4.7)	155.9 (5.9)	151.9 (3.8)	155.2 (4.8)	0.033	0.068
Weight (kg)	51.5 (6.7)	62.3 (9.6)	57.4 (7.9)	56.5 (6.3)	56.4 (6.8)	55.5 (5.8)	60.9 (10.2)	0.003	0.521
BMI (kg/m^2^)	20.4 (2.3)	24.7 (3.3)	23.9 (3.9)	22.9 (2.6)	23.2 (2.1)	24.1 (2.5)	25.3 (4.0)	< 0.001	0.344

Variables are presented as means (SD)

*BMI* Body mass index, *KL* Kellgren–Lawrence grade

### Reliability

The results of the intra- and inter-rater correlation coefficients, SEM, and MDC95 are presented in Table 2. The ICCs for all measures were relatively high.

**Table 2:** Intra-rater correlation coefficient and the inter-rater correlation coefficient

Variable of IFP	Subject	Model	ICC	95%CI	df	SEM	MDC_95_
Fiber orientation	Healthy adults	ICC (1.3)	0.88	0.61–0.96	9.0	0.01	0.04
		ICC (2.3)	0.84	0.49–0.96	9.0	0.02	0.05
	Patients with KOA	ICC (1.3)	0.88	0.67–0.96	9.0	0.01	0.04
		ICC (2.3)	0.84	0.57–0.95	9.0	0.02	0.05
Proportion of high-intensity area (%)	Healthy adults	ICC (1.3)	0.90	0.66–0.97	9.0	1.28	3.54
		ICC (2.3)	0.87	0.58–0.97	9.0	1.58	4.36
	Patients with KOA	ICC (1.3)	0.88	0.68–0.96	9.0	1.48	4.10
		ICC (2.3)	0.87	0.66–0.96	9.0	1.53	4.23
Movement of superficial part (cm/sec)	Healthy adults	ICC (1.3)	0.79	0.39–0.94	9.0	0.04	0.11
		ICC (2.3)	0.77	0.29–0.94	9.0	0.04	0.12
	Patients with KOA	ICC (1.3)	0.83	0.56–0.94	9.0	0.04	0.10
		ICC (2.3)	0.77	0.43–0.92	9.0	0.04	0.12
Movement of deep part (cm/sec)	Healthy adults	ICC (1.3)	0.74	0.28–0.92	9.0	0.05	0.15
		ICC (2.3)	0.70	0.15–0.92	9.0	0.06	0.16
	Patients with KOA	ICC (1.3)	0.76	0.41–0.92	9.0	0.05	0.14
		ICC (2.3)	0.76	0.42–0.92	9.0	0.05	0.15

*IFP* Infrapatellar fat pad, *ICC* (1.3) Intra-rater correlation coefficient, *ICC* (2.3) Inter-rater correlation coefficient, *SEM* Standard Error of the Measurement, *MDC* Minimal Detectable Change

### Between-group ultrasonographic analysis

**Fig. 3:**
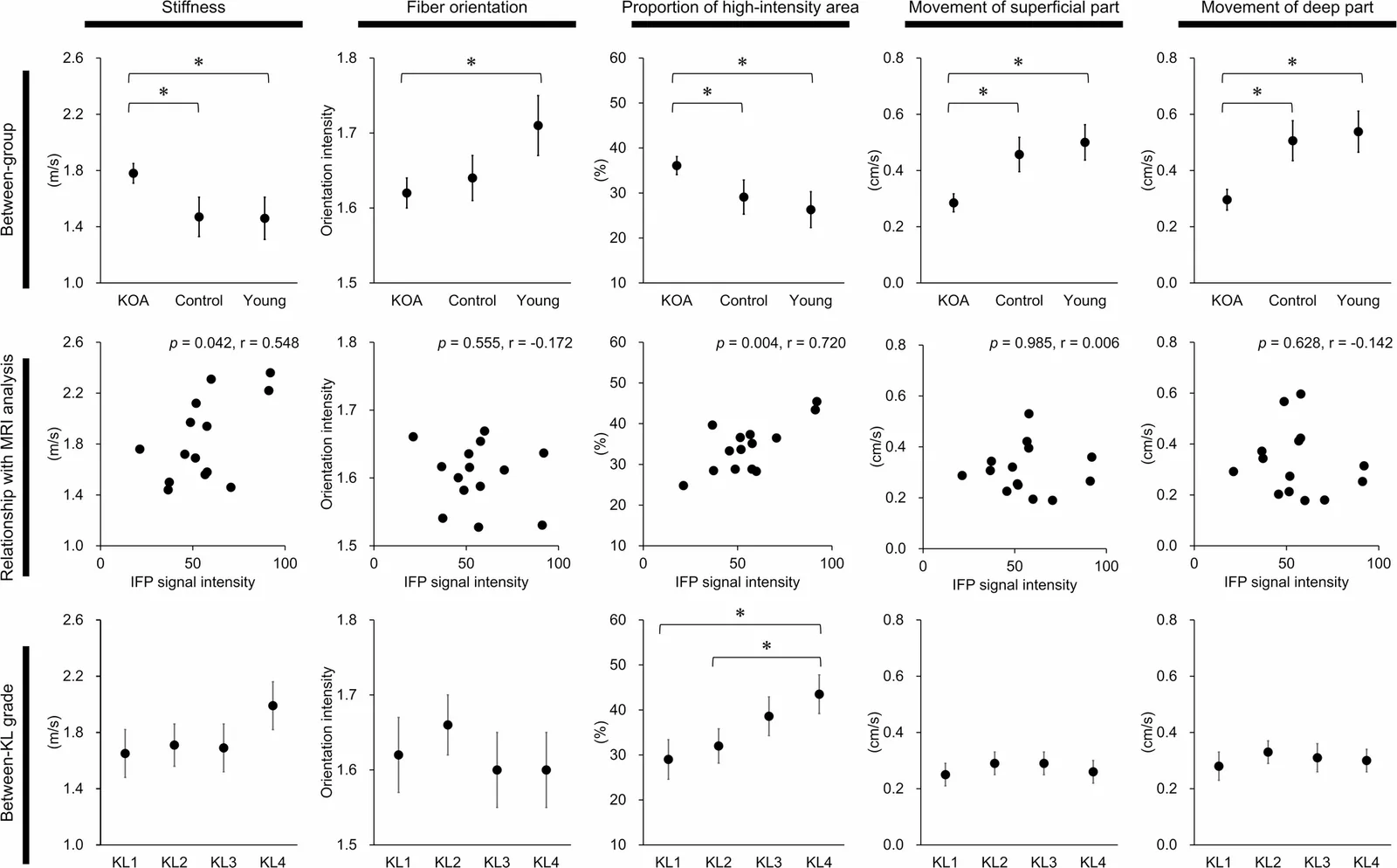
Comprehensive ultrasonographic and MRI evaluations of the IFP across clinical groups and disease severities. (Top row) Comparisons of adjusted means for IFP stiffness, fiber orientation, proportion of high-intensity area, and movement (superficial and deep parts) among the knee osteoarthritis (KOA), control, and young age groups. Error bars represent 95% confidence intervals (CIs). Asterisks (*) indicate significant differences between groups based on post hoc analyses with Bonferroni correction (*p* < 0.01). (Middle row) Scatter plots demonstrating the relationships between the IFP signal intensity on MRI and each ultrasonographic parameter within the KOA group. Spearman’s rank correlation coefficients (r) and exact p-values are presented for each plot. A p-value of < 0.01 was considered statistically significant for the MRI correlation analyses after Bonferroni correction. (Bottom row) Comparisons of the adjusted means for each ultrasonographic parameter across Kellgren–Lawrence (KL) grades I to IV within the KOA group. Error bars represent 95% CIs. Asterisks (*) indicate significant differences between grades based on post hoc analyses with Bonferroni correction (*p* < 0.01). IFP, infrapatellar fat pad; KOA, knee osteoarthritis; KL, Kellgren–Lawrence; MRI, magnetic resonance imaging; CI, confidence interval

The IFP stiffness showed significant main effects among groups (Supplementary Table 1). Post hoc analyses revealed significantly higher IFP stiffness in the KOA group compared to that in the control (MD 95% CI [0.152, 0.469]; *p* < 0.001) and young age groups(MD 95% CI [0.156, 0.493]; *p* < 0.001) (Fig. 3; Table 3).

**Table 3:** Unadjusted and adjusted post-hoc pairwise comparisons of ultrasonographic parameters

Variable	Group/KL grade	Adjusted Mean [95% CI]	Post-hoc comparisons	Model 1		Model 2	
				95% CI	*p*-value	95% CI	*p*-value
Between-group							
Stiffness (m/s)	KOA (*n* = 50)	1.780 [1.710, 1.860]	KOA vs. Control	[0.151, 0.465]	< 0.001	[0.152, 0.469]	< 0.001
	Control (*n* = 14)	1.470 [1.330, 1.610]	KOA vs. Young	[0.181, 0.487]	< 0.001	[0.156, 0.493]	< 0.001
	Young (*n* = 15)	1.460 [1.310, 1.600]	Control vs. Young	[-0.167, 0.219]	0.962	[-0.198, 0.226]	0.991
Fiber orientation	KOA (*n* = 50)	1.620 [1.600, 1.640]	KOA vs. Control	[-0.067, 0.016]	0.449	[-0.067, 0.017]	0.473
	Control (*n* = 14)	1.640 [1.610, 1.680]	KOA vs. Young	[-0.126, -0.045]	< 0.001	[-0.132, -0.043]	< 0.001
	Young (*n* = 15)	1.710 [1.670, 1.750]	Control vs. Young	[-0.111, -0.009]	0.059	[-0.119, -0.006]	0.076
Proportion of high-intensity area (%)	KOA (*n* = 50)	36.100 [34.100, 38.100]	KOA vs. Control	[2.968, 11.452]	0.003	[2.737, 11.263]	0.005
	Control (*n* = 14)	29.100 [25.300, 32.900]	KOA vs. Young	[4.847, 13.093]	< 0.001	[5.278, 14.322]	< 0.001
	Young (*n* = 15)	26.300 [22.300, 30.200]	Control vs. Young	[-3.458, 6.978]	0.779	[-2.897, 8.497]	0.592
Movement of superficial part (cm/s)	KOA (*n* = 50)	0.285 [0.253, 0.317]	KOA vs. Control	[-0.235, -0.098]	< 0.001	[-0.240, -0.104]	< 0.001
	Control (*n* = 14)	0.457 [0.396, 0.518]	KOA vs. Young	[-0.304, -0.171]	< 0.001	[-0.287, -0.143]	< 0.001
	Young (*n* = 15)	0.500 [0.437, 0.563]	Control vs. Young	[-0.155, 0.013]	0.216	[-0.133, 0.048]	0.621
Movement of deep part (cm/s)	KOA (*n* = 50)	0.296 [0.259, 0.333]	KOA vs. Control	[-0.283, -0.123]	< 0.001	[-0.290, -0.131]	< 0.001
	Control (*n* = 14)	0.506 [0.435, 0.577]	KOA vs. Young	[-0.350, -0.194]	< 0.001	[-0.326, -0.158]	< 0.001
	Young (*n* = 15)	0.538 [0.465, 0.611]	Control vs. Young	[-0.168, 0.029]	0.344	[-0.137, 0.074]	0.824
Between-KL grade							
Proportion of high-intensity area (%)	KL I (*n* = 12)	29.000 [24.600, 33.400]	Ⅰ vs. Ⅱ	[-8.055, 3.015]	0.796	[-8.593, 2.573]	0.700
	KL II (*n* = 14)	32.000 [28.200, 35.800]	Ⅰ vs. Ⅲ	[-13.858, -2.102]	0.042	[-16.070, -3.090]	0.024
	KL III (*n* = 11)	38.600 [34.300, 42.900]	Ⅰ vs. Ⅳ	[-17.696, -6.424]	< 0.001	[-21.121, -7.859]	< 0.001
	KL IV (*n* = 13)	43.500 [39.200, 47.700]	Ⅱ vs. Ⅲ	[-11.136, 0.216]	0.227	[-12.465, -0.695]	0.126
			Ⅱ vs. Ⅳ	[-14.955, -4.125]	0.005	[-17.425, -5.535]	0.002
			Ⅲ vs. Ⅳ	[-9.837, 1.677]	0.491	[-10.674, 0.854]	0.328

Data are presented as adjusted means [95% CI] for Model 2. Model 1 represents the unadjusted (raw) analysis. Model 2 represents the adjusted analysis using ANCOVA. For between-group comparisons, Model 2 was adjusted for BMI, with maximum IQE pressure additionally included for movement analyses. For between-KL grade comparisons, Model 2 was adjusted for age and BMI, with maximum IQE pressure additionally included for movement analyses

*IFP* Infrapatellar fat pad, *KOA* Knee osteoarthritis, *KL* Kellgren-Lawrence, *CI* Confidence interval.

The IFP fiber orientation was higher in the young age group than in the KOA group (MD 95% CI [-0.132, -0.043]; *p* < 0.001) and the control group (MD 95% CI [-0.119, -0.006]; *p* = 0.076, not significant after Bonferroni correction) (Fig. 3; Table 3).

The IFP proportion of high-intensity area in the KOA group was significantly higher than in the control (MD 95% CI [2.737, 11.263]; *p* = 0.005) and young age groups (MD 95% CI [5.278, 14.322]; *p* < 0.001) (Fig. 3; Table 3).

Regarding IFP movement, separate analyses for both regions demonstrated significant main effects (Supplementary Table 1). The superficial movement in the KOA group was significantly lower than in the control (MD 95% CI [-0.240, -0.104]; *p* < 0.001) and young age groups (MD 95% CI [-0.287, -0.143]; *p* < 0.001) (Fig. 3; Table 3). Similarly, the deep movement in the KOA group was significantly lower than in the control (MD 95% CI [-0.290, -0.131]; *p* < 0.001) and young age groups (MD 95% CI [-0.326, -0.158]; *p* < 0.001) (Fig. 3; Table 3).

### Relationship between ultrasound and MRI imaging analysis

Of the 50 knees with KOA, MRI data were available for 14 knees and were included in the MRI correlation analysis. A comparison of baseline characteristics and ultrasound variables between patients with KOA with (*n* = 14) and without (*n* = 36) MRI data showed no significant differences (Supplementary Table 2). The mean MRI signal intensity of the IFP in the KOA was 55.613 (95% CI [44.819, 66.406]). The IFP signal intensity on MRI had a positive correlation with the IFP proportion of high-intensity area (*r* = 0.720, *p* = 0.004, 95% CI [0.307, 0.905]) (Fig. 3). Other associations did not reach the Bonferroni-adjusted significance threshold, including those with IFP stiffness (*r* = 0.548, *p* = 0.042, 95% CI [0.025, 0.836]) or IFP fiber orientation (*r* = -0.172, *p* = 0.555, 95% CI [-0.644, 0.394]) or IFP movement in either the superficial (*r* = 0.006, *p* = 0.985, 95% CI [-0.527, 0.535]) or deep parts (*r* = -0.142, *p* = 0.628, 95% CI [-0.626, 0.420]) (Fig. 3).

### Ultrasound imaging analysis of the between-KL grade difference

The IFP proportion of high-intensity area showed a significant main effect of KL grade (Supplementary Table 1). Post hoc analyses indicated that the proportion of high-intensity area in KL grade IV (95% CI [39.200, 47.700]) was significantly higher than in KL grade I (95% CI [24.600, 33.400]) and KL grade II (95% CI [28.200, 35.800]) (Fig. 3; Table 3). There were no significant main effects of KL grade on IFP stiffness, fiber orientation, superficial movement, or deep movement after Bonferroni correction (Supplementary Table 1, Table 3). The proportion of high-intensity area between KL grade III and the other grades did not differ significantly.

## Discussion

This study revealed three main findings. First, compared with the control and young age groups, patients with KOA exhibited significantly higher IFP stiffness and proportion of high-intensity area, along with considerably reduced IFP movement. Second, the proportion of high-intensity area, as measured using ultrasound, was significantly and positively correlated with IFP signal intensity on MRI after accounting for multiple comparisons. Third, the IFP proportion of high-intensity area was significantly higher in advanced KOA (KL grade IV) than in milder stages (KL grades I and II).

Previous ultrasonographic studies have quantitatively assessed individual IFP characteristics such as elasticity [14], echo intensity [19], and thickness changes [16–18]. However, these investigations typically focused on a single parameter (such as the relationship with pain or between-group differences). In contrast, this study provides a more comprehensive characterization of the IFP by simultaneously evaluating both fibrous structural (proportion of high-intensity area and fiber orientation) and mechanical properties (stiffness and movement). Increased IFP signal intensity on T2-weighted MRI primarily reflects inflammatory features, including edema, neovascularization, and synovitis [5, 10], which are associated with radiographic disease progression [7]. Although increased echogenicity on binarized ultrasound can result from fibrosis, it may also reflect inflammation, edema, or calcification. Therefore, the proportion of high-intensity area should be interpreted as an imaging parameter reflecting alteration in the overall tissue rather than as a direct histological measure of fibrosis. The positive correlation between MRI signal intensity and the proportion of high-intensity area suggests a potential pathophysiological link where chronic inflammation and edema occur concurrently with structural alterations in the IFP. An increase in the proportion of high-intensity area likely contributes to greater stiffness, which in turn may restrict IFP mobility. Interestingly, IFP fiber orientation and movement were not correlated with MRI signal intensity. These non-significant findings suggest that structural disorganization and functional mobility restriction might represent different biomechanical dimensions of IFP pathology that are not directly proportional to the degree of fluid-sensitive inflammatory signals. Alternatively, these could be attributed to a type II error resulting from the small sample size of the MRI subgroup.

These findings may help clarify the pathogenesis of the IFP in patients with KOA. The results of this study showed that the KOA group had increased IFP stiffness and proportion of high-intensity area, and decreased IFP movement, compared with the control group, whereas both the KOA and control groups demonstrated lower IFP fiber orientation than the young group. Given the cross-sectional study design, these findings cannot establish a temporal sequence of disease progression. However, they suggest that distinct IFP tissue alterations are associated with KOA. The decreased fiber orientation observed in both older groups, regardless of KOA, may reflect age-related remodeling of the extracellular matrix rather than a disease-specific pathology. Furthermore, the IFP proportion of high-intensity area increased with KOA severity, suggesting that this structural alteration may become more pronounced in advanced KOA.

Ultrasonography is a practical, non-invasive modality for the quantitative assessment of IFP pathology in KOA. This technique enables simultaneous evaluation of the fibrous structure and mechanical characteristics of the IFP, including proportion of high-intensity area, stiffness, and movement, in a clinical setting. The proportion of high-intensity area appears particularly promising as a quantitative measure of IFP tissue alteration and disease severity. This approach is analogous to established ultrasonographic techniques for evaluating structural changes in other soft tissues [32]. Consequently, routine ultrasonographic assessment of the IFP proportion of high-intensity area may be useful for developing and monitoring therapeutic interventions such as physical therapy and pharmacological treatment.

This study has some limitations. Sex-related differences in the studied population may have influenced the results, because only female participants were included to eliminate confounding effects, making it unclear whether the findings are generalizable to males. Additionally, we did not perform radiographic or MRI examinations in the control group, leaving the possibility of asymptomatic radiographic KOA. The severity of clinical symptoms was also not compared. Furthermore, although patients with inflammatory or rheumatologic diseases were clinically excluded, we did not perform laboratory assessments (e.g., blood tests) to definitively rule out subclinical inflammatory arthropathies, which can also cause increased T2 signals in the IFP. Furthermore, MRI signal intensity was derived from conventional fat-suppressed T2-weighted images and was not normalized to a reference tissue; therefore, it should be interpreted as a semi-quantitative measure, and absolute values may be influenced by acquisition-related factors. Finally, because performing MRI for all patients with KOA is not routinely indicated or cost-effective, MRI examinations were limited to 14 patients based on physician indication. This introduces a potential selection bias, as the patients who underwent MRI might have had more severe knee pain or symptoms. A single examiner performed the ultrasound assessments without being blinded to the participants’ group assignments, introducing a potential risk of observer bias. The pressure of the blood pressure cuff was selected as 100 mmHg, and while IFP stiffness and movement could potentially be influenced by changes in the knee joint angle caused by the pressure, the knee flexion angle was strictly maintained at 10° throughout the procedure. Therefore, the physical impact of the cuff on the measured IFP parameters is considered minimal. Additionally, the echo intensity obtained with ultrasound may vary across devices. Furthermore, the IFP proportion of high-intensity area and histological findings were not directly compared; thus, without histological validation, increased echogenicity cannot be definitively interpreted as fibrosis. In terms of statistical analysis, because we applied a strict Bonferroni correction for multiple comparisons, the study may have been underpowered to detect differences in non-significant comparisons, increasing the risk of type II errors. Finally, when considering errors in measurement, the absolute lower bounds of the 95% confidence intervals for between-group differences in the proportion of high-intensity area and IFP movement included values slightly smaller than their respective MDC95 values; thus, measurement error could not be completely ruled out.

## Conclusion

Patients with KOA demonstrated increased IFP stiffness and proportion of high-intensity area. They also demonstrated decreased IFP movement on ultrasound compared with the control group, indicating a distinct IFP fibrous structure and mechanical characteristics in KOA. Notably, the proportion of high-intensity area was significantly higher in advanced KOA (KL grade IV) than in milder grades (I and II), and correlated with MRI signal intensity, suggesting its potential utility as a quantitative imaging parameter for assessing IFP tissue alterations.

## Supplementary Information

Supplementary Material 1: Supplementary Table 1. Results of ANCOVA for IFP parameters. Supplementary Table 2. Comparison of baseline characteristics and ultrasound-derived parameters between patients with KOA with and without MRI data.

## Data Availability

The data supporting the findings of this study are available from the corresponding author upon reasonable request.

## Abbreviations

IFP: Infrapatellar fat pad
KOA: Knee osteoarthritis
US: Ultrasonography
MRI: Magnetic resonance imaging
KL: Kellgren–Lawrence
PIV: Particle image velocimetry
IQE: Isometric quadriceps exercise
ANCOVA: Analysis of covariance
ICC: Intra-class correlation coefficient
SEM: Standard error of the measurement
MDC95: Minimal detectable change at the 95% confidence interval
MD: Mean difference
CI: Confidence interval
