# Supplementary material for: Ultrasonographic evaluation of fibrous structure and mechanical characteristics of the infrapatellar fat pad in female patients with knee osteoarthritis: a cross-sectional study
Source: BMC Musculoskelet Disord. 2026 Jul 23;27:885. doi: 10.1186/s12891-026-10250-z (PMC13681884; doi:10.1186/s12891-026-10250-z)
Supplement: Supplementary file 1 [file 12891_2026_10250_MOESM1_ESM.docx]

**Additional File 1**

Supplementary Table 1. Results of ANCOVA for IFP parameters

| Dependent variable | Fixed effects | Model 1 |  |  |  | Model 2 |  |  |  |
| --- | --- | --- | --- | --- | --- | --- | --- | --- | --- |
|  |  | F value | df | P value | η^2^ | F value | df | P value | η^2^ |
| Stiffness (m/s) | Group | 14 | 2 | <.001 | 0.269 | 12.894 | 2 | <.001 | 0.256 |
|  | KL grade | 2.89 | 3 | 0.046 | 0.159 | 3.221 | 3 | 0.032 | 0.18 |
| Fiber orientation | Group | 8.77 | 2 | <.001 | 0.188 | 7.746 | 2 | <.001 | 0.171 |
|  | KL grade | 1.94 | 3 | 0.136 | 0.112 | 1.713 | 3 | 0.178 | 0.105 |
| Proportion of high-intensity area (%) | Group | 12.4 | 2 | <.001 | 0.247 | 12.725 | 2 | <.001 | 0.253 |
|  | KL grade | 7.59 | 3 | <.001 | 0.331 | 7.471 | 3 | <.001 | 0.337 |
| Movement of superficial part (cm/s) | Group | 30.9 | 2 | <.001 | 0.448 | 26.14 | 2 | <.001 | 0.411 |
|  | KL grade | 1.02 | 3 | 0.391 | 0.063 | 0.784 | 3 | 0.509 | 0.051 |
| Movement of deep part (cm/s) | Group | 30.7 | 2 | <.001 | 0.447 | 26.26 | 2 | <.001 | 0.412 |
|  | KL grade | 1.26 | 3 | 0.3 | 0.071 | 1.33 | 3 | 0.277 | 0.08 |

Data are presented as the results of ANCOVA. Model 1 represents the unadjusted (raw) analysis. Model 2 represents the adjusted analysis. For between-group comparisons, Model 2 was adjusted for BMI. Maximum IQE pressure was included as a covariate for superficial and deep movement. For between-KL grade comparisons, Model 2 was adjusted for age, BMI. Maximum IQE pressure was included as a covariate for superficial and deep movement. IFP, infrapatellar fat pad; KL, Kellgren–Lawrence; df, degrees of freedom; η^2^, partial eta-squared.

Supplementary Table 2. Comparison of baseline characteristics and ultrasound-derived parameters between patients with KOA with and without MRI data

|  | KOA group with MRI data | KOA group without MRI data | Mean difference | 95% CI | p-value |
| --- | --- | --- | --- | --- | --- |
| Age (years) | 67.29 (11.21) | 66.28 (11.79) | -1.01 | [-8.38, 6.36] | 0.785 |
| Height (cm) | 156.07 (4.71) | 154.69 (5.52) | -1.38 | [-4.74, 1.99] | 0.415 |
| Weight (kg) | 58.00 (8.26) | 57.13 (7.91) | -0.87 | [-5.94, 4.20] | 0.731 |
| BMI (kg/m2) | 23.79 (3.04) | 23.88 (3.10) | 0.09 | [-1.87, 2.04] | 0.930 |
| K-L grade | 2.57 (1.09) | 2.47 (1.16) | -0.1 | [-0.82, 0.62] | 0.784 |
| Stiffness (m/s) | 1.83 (0.32) | 1.77 (0.31) | -0.07 | [-0.26, 0.13] | 0.506 |
| Fiber orientation | 1.61 (0.05) | 1.63 (0.09) | 0.02 | [-0.03, 0.07] | 0.404 |
| Proportion of high-intensity area (%) | 34.31 (6.06) | 36.62 (8.82) | 2.32 | [-2.85, 7.49] | 0.372 |
| Movement of superficial part (cm/s) | 0.31 (0.09) | 0.27 (0.08) | -0.04 | [-0.09, 0.01] | 0.142 |
| Movement of deep part (cm/s) | 0.33 (0.13) | 0.28 (0.08) | -0.05 | [-0.12, 0.01] | 0.096 |

Data are presented as mean (standard deviation). Differences between the two groups were analyzed using the independent t-test. KOA: knee osteoarthritis; MRI: magnetic resonance imaging; BMI: body mass index; K-L: Kellgren–Lawrence; CI: confidence interval.
